# Emerging Role of CREB in Epithelial to Mesenchymal Plasticity of Pancreatic Cancer

**DOI:** 10.3389/fonc.2022.925687

**Published:** 2022-06-21

**Authors:** Siddharth Mehra, Samara Singh, Nagaraj Nagathihalli

**Affiliations:** ^1^ Division of Surgical Oncology, Department of Surgery, University of Miami Miller School of Medicine, Miami, FL, United States; ^2^ Sylvester Comprehensive Cancer Center, University of Miami, Miami, FL, United States

**Keywords:** pancreatic cancer, CREB, RAS, epithelial to mesenchymal plasticity, MicroRNAs, metastasis, therapeutic resistance

## Abstract

Pancreatic ductal adenocarcinoma (PDAC) is an aggressive solid malignancy with a high rate of metastasis and therapeutic resistance as its major hallmarks. Although a defining mutational event in pancreatic cancer initiation is the presence of oncogenic *KRAS*, more advanced PDAC lesions accumulate additional genomic alterations, including loss of tumor suppressor gene *TP53.* Co-occurrence of mutant *KRAS* and *TP53* in PDAC promotes hyperactivation of cancer cell signaling pathways driving epithelial to mesenchymal plasticity (EMP). The cellular process of EMP influences the biological behavior of cancer cells by increasing their migratory and invasive properties, thus promoting metastasis. Our previous work has demonstrated that oncogenic KRAS-mediated activation of cyclic AMP response element-binding protein 1 (CREB) is one of the critical drivers of PDAC aggressiveness. The therapeutic approach of targeting this key transcription factor attenuates tumor burden in genetically engineered mouse models (GEMMs) of this disease. Herein, we discuss the significant role of CREB in perpetuating disease aggressiveness and therapeutic resistance through the EMP process. Furthermore, this review updates the therapeutic implications of targeting CREB, highlighting the challenges and emerging approaches in PDAC.

## Introduction

Pancreatic ductal adenocarcinoma (PDAC) is an aggressive solid malignancy associated with significant mortality and is projected to be the second leading cause of cancer-related deaths by 2030 (1, 2). Delayed diagnosis, lack of effective treatments, and high metastatic propensity put this disease in the category of cancers with an extremely poor 5-year survival. PDAC originates from normal pancreatic epithelium transitioning to a neoplastic precursor state known as a pancreatic intraepithelial neoplasm (PanIN), instigating the oncogenic transformation into a ductal adenocarcinoma (3). This gradual progression towards invasive cancer is supported by a unique dependency on the mutated *KRAS* oncogene, prevalent in more than 90% of PDAC patients. Other than the presence of *KRAS*, inactivating mutation in *TP53* also co-occur in more than 70% of PDAC patients. The underlying cooperativity between these two key dominant oncogenic drivers promotes PDAC progression and contributes to metastatic dissemination of this disease (4–7). Therefore, a major unmet need is to understand the cellular mechanisms responsible for promoting disease aggressiveness and to further identify actionable therapeutic strategies to improve the prognosis of this malignancy (8).

**Figure 1:**
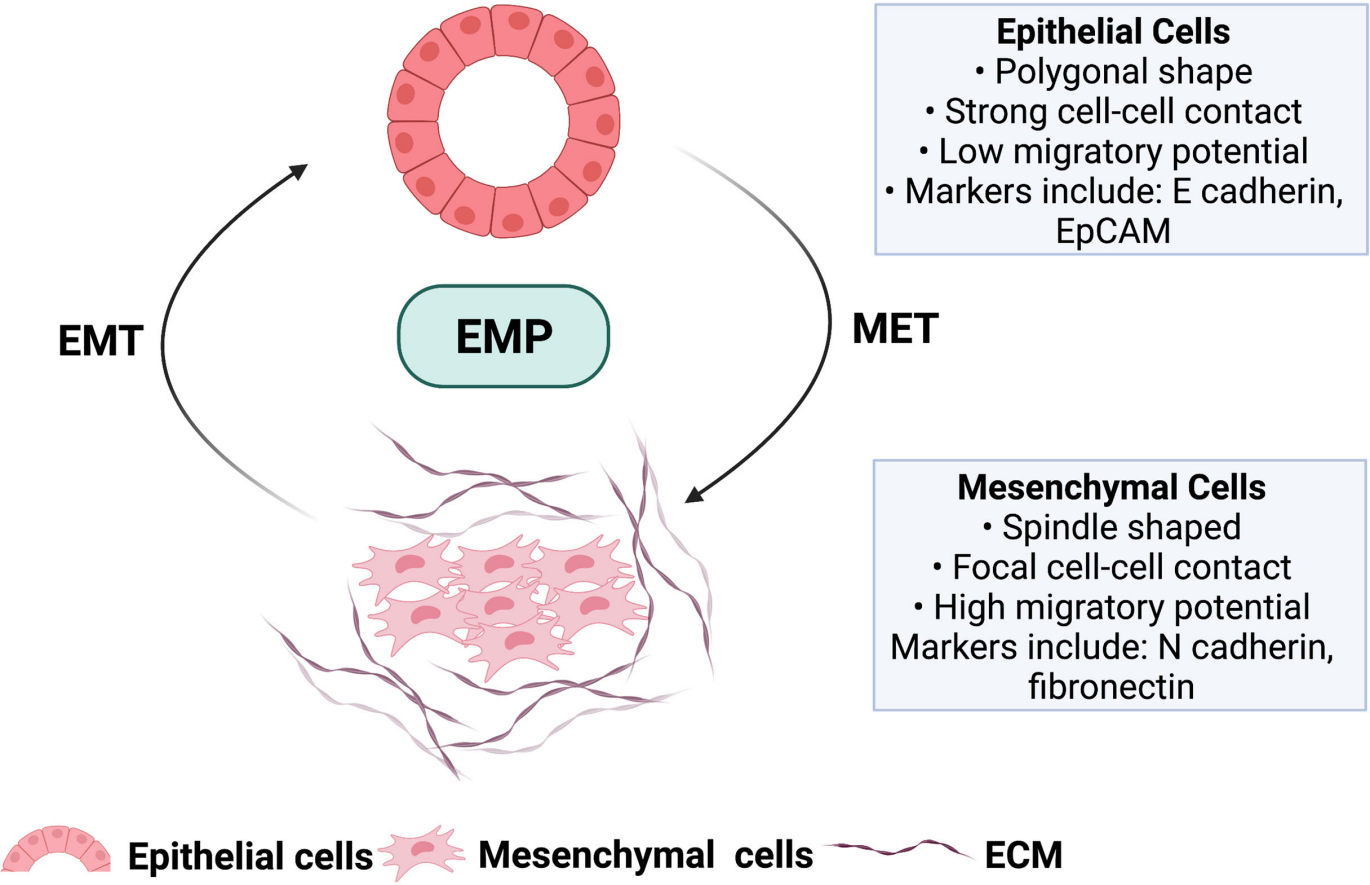
Hallmark features of malignant epithelial cells undergoing forward differentiation towards mesenchymal cell type (EMT) and the reversal mesenchymal to epithelial transition (MET). These two biological processes give rise to the concept of epithelial to mesenchymal plasticity (EMP) involved in tumor progression and metastasis. ECM; Extracellular matrix. Image created with BioRender.com (Agreement number TL23SM1HJO).

The presence of dominant genomic alterations modulates several oncogenic cellular signaling events in PDAC, which enables tumor cells to exhibit a distinctive cellular plasticity, enabling them to transform into an invasive and migratory mesenchymal phenotype, in a process known as epithelial to mesenchymal transition (EMT) and subsequent reversal from mesenchymal to epithelial transition (MET). These two biological phenomena give rise to the concept of epithelial-mesenchymal plasticity (EMP) **(** **Figure 1** **)**. This cellular transformation of EMP in cancer cells leads to the disruption of tissue homeostasis and facilitates crosstalk between different stromal components within the tumor microenvironment (TME), contributing to intratumoral heterogeneity (9).

Cyclic AMP response element-binding protein 1 (CREB) is a transcriptional coactivator that has been shown to be activated downstream of Ras-dependent oncogenic signaling pathways (10, 11). Once activated through phosphorylation at Ser133, CREB binds to its coactivator, the CREB-binding protein (CBP), enabling the recruitment of additional transcriptional machinery elements necessary to drive transcriptional programs of malignant progression (12). The present review will focus on the various mechanisms of CREB-dependent EMP, downstream of mutant *KRAS* in PDAC and other cancer types. Additionally, the therapeutic potential and challenges of targeting CREB in the attenuation of cellular plasticity and overcoming drug resistance will be examined. Overall, identifying novel molecular targets to attenuate the cellular process of EMP may improve clinical outcomes in patients with PDAC.

## EMP and Metastasis in PDAC

Mortalities associated with PDAC do not occur due to the primary tumor but are often found to be associated with the metastatic dissemination of tumor cells, which begins early in most patients (2, 13). More than 90% of the patients diagnosed display local or distant metastatic disease. This rapid progression associated with this malignancy warrants new studies on the key cellular processes driving this metastatic behavior (8). Mechanistically, PDAC cells exhibit the unique hallmark of EMT, displaying higher invasive characteristics, cancer stem cell-like behavior, and greater resistance to therapies (14). During this forward differentiation process, they lose the expression of epithelial markers (including E cadherin, occludin, claudin, and laminin) and gain mesenchymal phenotypic plasticity with elevated expression of markers such as N-cadherin, vimentin, and fibronectin (13, 15, 16). These changes are often associated with the activation of EMT-driven transcriptional programs led by transcription factors such as TWIST, SNAIL, and ZEB, which coordinatively repress E-cadherin levels while promoting expression of mesenchymal differentiation markers. Once these invading cancer cells colonize the metastatic sites, they undergo the reverse EMT process known as MET, which helps them to adopt a high proliferation rate in the invaded TME (17–19). Taken together, these two distinct but related cellular programs- EMT, and the reversal mesenchymal-epithelial transition MET, play a significant role in PDAC tumorigenesis. EMP mediated cellular plasticity allows PDAC tumor cells to detach and migrate from their site of origin (invasion) and gain access to lymphatic blood vasculature and distant sites (extravasation), to form metastases (20). Therefore, understanding the molecular regulation of these steps can help to elucidate therapeutic options to restrict EMP-mediated tumor metastases in PDAC.

## *RAS/TP53* and EMP in PDAC

In mutant *KRAS/TP53-*driven tumors, including PDAC, these cellular plasticity programs and tumorigenesis are interconnected; this was established using genetically engineered mouse models (GEMMs) of PDAC, where tumor cells harboring these oncogenic mutation displayed EMT like features at an early stage after tumor initiation (13). Additionally, the presence of soluble ligands, including growth factors mediating activation of receptor tyrosine kinases (RTKs), also drives EMP programs in PDAC in a RAS-dependent manner (21, 22). Shedding of EGFR ligands, including amphiregulin from PDAC cells, results in an autocrine feedback loop to further promote the KRAS hyperactivation (21–23). Once activated, signaling networks downstream of KRAS, including MEK/ERK and PI3K/AKT, can promote EMP. Targeted inhibition of these downstream effector kinases has been shown to reverse KRAS-mediated epithelial plasticity (24). Transcriptomics-based gene set enrichment analysis (GSEA) of highly metastatic cell lines derived from KPC (*LSL-Kras^G12D/+^;Trp53^R273H/+^;Pdx1^Cre/+^*) GEMM of PDAC identified significant enrichment of KRAS-dependent gene signatures compared to cell clones with low metastatic potential (25). Several studies over recent years have established the vast contribution of various molecular regulators and downstream KRAS effectors to perpetuate PDAC aggressiveness. Direct targeting of KRAS has largely been unsuccessful; therefore, efforts have now shifted to targeting its downstream effectors (26–28), providing opportunities for novel therapeutic interventions in PDAC.

## CREB Activation in PDAC

**Figure 2:**
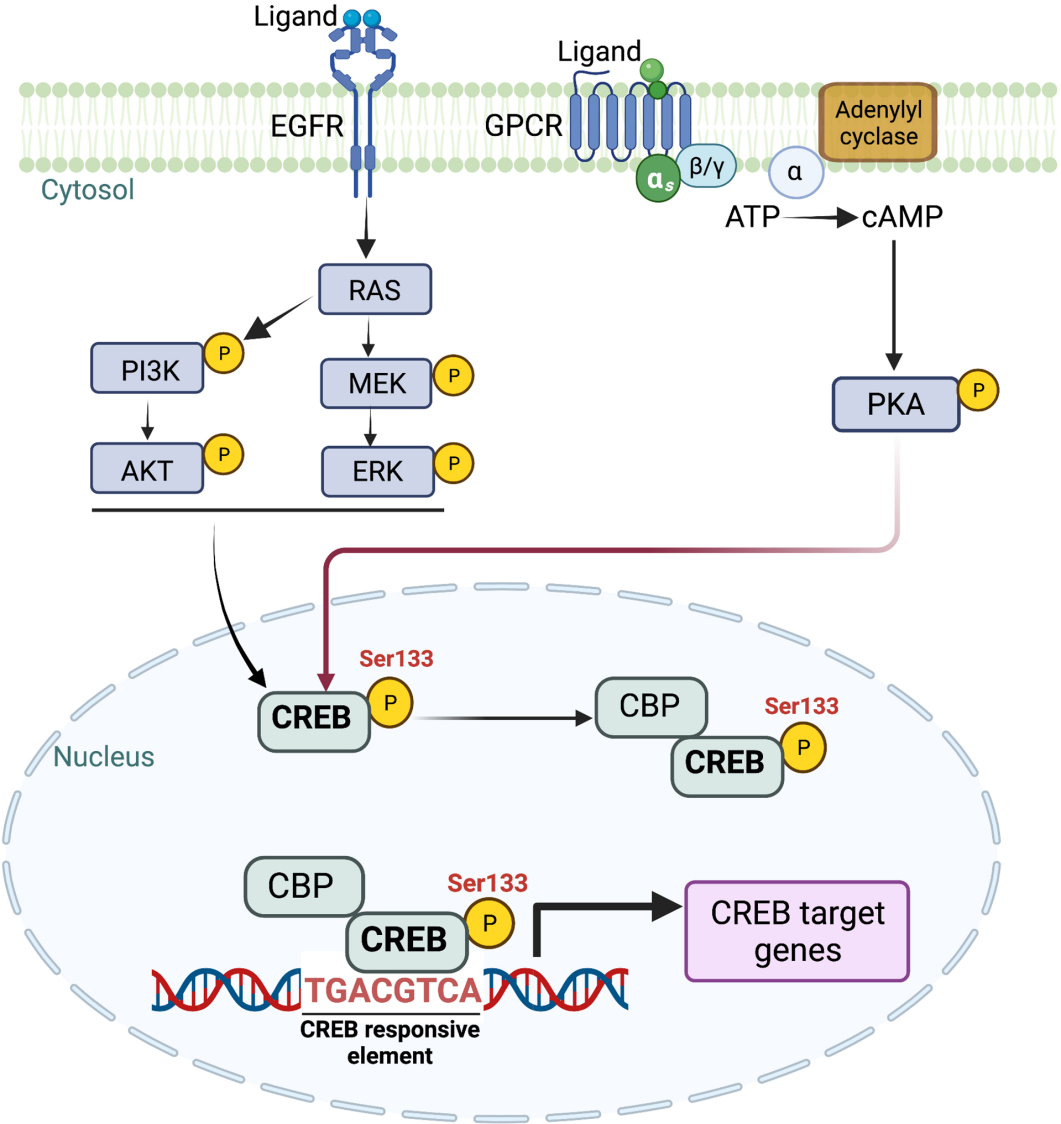
Schematic representation of multiple upstream signaling cascades involved in intracellular CREB activation. CREB, once phosphorylated at Ser 133, translocates to the nucleus. Once translocated, CREB binds to CRE (cAMP response element) and provides transcriptional ability to activate multiple downstream targets. Image created with BioRender.com (Agreement number ZW23SM1LR3).

Targeting individual KRAS-mediated factors may provide therapeutic insights and understanding the cooperative interactions between different transcriptional and signaling networks remains an essential need to target the heterogenous tumor cell population of KRAS-driven PDAC effectively. With long-standing efforts of our laboratory in deciphering the molecular underpinnings of KRAS-mediated signaling pathways in the pathogenesis of PDAC, our work has demonstrated that oncogenic KRAS activates a master transcription regulator CREB (11). We have uncovered its role as a critical driver of PDAC aggressiveness, and its overexpression is associated with poor prognosis in the patients. Activation of CREB is mediated through MEK and AKT-dependent signaling pathways in KRAS mutant PDAC **(** **Figure 2** **)** (11). Additional work from our lab by Srinivasan et al. further illuminated GM-CSF-mediated CREB activation as a critical driver of the development and progression of smoking-associated PDAC tumorigenesis to promote disease aggressiveness. Therapeutic targeting of CREB significantly attenuated tumor burden in our PDAC disease model (29). Taken together, it is vital to understand CREB’s function and its role in EMP regulation in this disease.

## CREB Structure and Function

**Figure 3:**
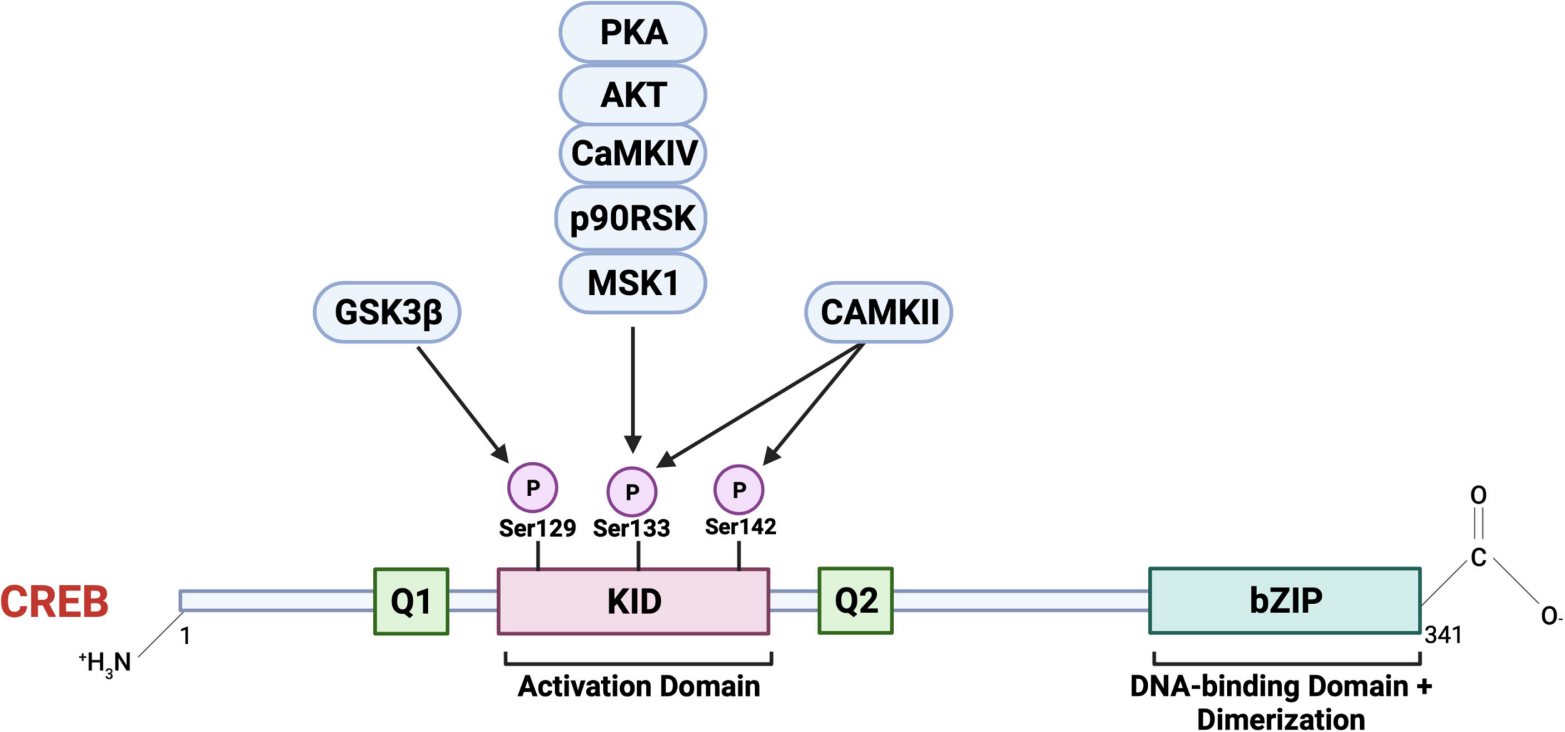
The primary structure of CREB consists of an N terminal containing an activation domain and a smaller C terminal with a basic region/leucine zipper (bZIP) DNA-binding and dimerization domain. CREB also contains kinase inducible domain (KID) and hydrophobic glutamate-rich domains (Q1 and Q2). A critical event involving CREB activation is the phosphorylation at Ser 133 in KID by multiple upstream cellular effector kinases. CREB, once activated, acts as a master transcriptional regulator of multiple downstream cellular targets. Image created with BioRender.com (Agreement number NZ23SM1ZAY).

CREB1 was the first reported basic leucine zipper family transcription factor whose activity was shown to be regulated by auto-phosphorylation. This was found to be a shared feature among its family members, including activating transcription factor (ATF1) and cAMP response element modulator (CREM) (30). CREB, once translated into protein, constitutes 341 amino acids in length, forming a 37 kDa transcription factor (31). The protein structure of CREB consists of an NH_2_-terminal activation domain, COOH-terminal basic region/leucine zipper domain (bZIP), DNA-binding, and dimerization domain (32). The primary structure **(** **Figure 3** **)** of the CREB has a centrally located 60-amino-acid kinase-inducible domain (KID), which comprises multiple phosphorylation sites. Hydrophobic glutamine-rich domains (Q1 and Q2) are present on either side of the KID. Q1 is a basal transcriptional activation domain involved in the interaction of CREB with TATA-binding protein to regulate gene expression. In contrast, the other Q2 domain is responsible for interaction with RNA polymerase II transcriptional initiation complex leading to recognition and binding of CREB to its canonical CREB responsible element (CRE) sequence, 5’- TGACGTCA-3’ (32, 33). Furthermore, the bZIP dimerization domain at the carboxy-terminal is involved in its dimerization and is required for CREB binding to DNA regulatory sequences (34).

Activation of CREB involves reversible phosphorylation at numerous serine residues positioned at 129 (S129), 133 (S133), and 142 (S142). This phosphorylation event is triggered *via* multiple cellular effector kinases in response to growth factors or extracellular stress stimuli (35, 36). Some of the critical upstream activators involved in CREB phosphorylation, and its activation include protein kinase A (PKA), protein kinase B (PKB/AKT), the mitogen-activated kinase (MAPK), 90 kDa ribosomal S6 kinase (RSK), AKT, protein kinase C (PKC), calcium/calmodulin-dependent protein kinase II (CaMKII) (10, 35–37). The significance of phosphorylation at the position Ser 133 is extensively studied within the context of CREB activation and mediating its downstream effects on target genes. In response to intracellular signals, including cAMP, induces recruitment of CBP, a transcriptional co-activator, and its paralogue p300 (37), together this complex is active and promotes nuclear gene expression *via* targeting various promoter regions containing the highly conserved cAMP-responsive element (CRE) TGACGTCA (38–40). Some CREB target genes contain CRE and half-CRE elements (TGACG/CGTCA), and binding is dependent on the TATA box in their promoters. Out of the 4000 identified promoter regions with a predicted solid CREB binding affinity, only around 339 genes contain complete CRE and TATA boxes, and less than 100 genes have been validated as direct CREB targets in a cAMP-responsive manner (41). This suggests the role of CREB as a transcriptional regulator independent of the presence of CRE.

With the advancements of high throughput technologies in the field of cancer signaling and therapeutics, compelling scientific evidence demonstrates that CREB and CREB-regulated gene targets play an essential role in promoting tumor initiation, progression, and aggressiveness, implicating CREB as a critical transcription factor with proto-oncogene characteristics across multiple cancer types (42–47). In addition the role of CREB in the regulating protein-coding genes involved in tumorigenesis, several genomic-wide studies have also identified CREB-dependent regulation of non-coding genes (microRNAs), specifically within the context of EMP mediated tumor growth and aggressiveness.

## CREB-microRNA(miRNAs) and EMP

The cellular process of EMP during carcinogenesis is regulated by activating multiple transcription factors, including TWIST, SNAIL, and ZEB1, which are involved in modulating the expression of several cell adhesion and tight junction proteins (48). Studies highlighting the direct involvement of CREB in regulating the expression of genes involved in epithelial cell plasticity are limited. However, it still plays a pivotal role in regulating an EMP program by interacting with multiple microRNAs (miRNAs). miRNAs are small non-coding RNAs constituted of 18-22 nucleotides and are involved in regulating gene expression by binding to the 3’UTR of their target mRNA transcript (49–51). Mounting scientific evidence recently suggests that CREB transcriptionally regulates the expression of multiple miRNAs; additionally, CREB expression itself can also be modulated by miRNAs, thus forming a feedback loop.

The CREB-miRNA axis has been found to play a crucial role in influencing the tumorigenic potential of cancer cells as well as in EMP-mediated metastasis. Within the context of PDAC, Zhang et al. demonstrated that intracellular activation of zinc-dependent transcription factor (ZIP-4) promotes CREB activation, which upregulates transcription of miRNA-373 to influence PDAC tumor growth both *in vitro* and *in vivo* (52). Additionally, in bladder cancer, CREB regulation by miRNA-433 alters the EMT potential of tumor cells by targeting the c-Met/AKT/GSK-3β/SNAIL signaling pathway (53). The strong association of the CREB transcription factor promoting miRNAs in the development and progression of gastric cancer metastasis was reported by Liu et al., where overexpression of CREB was associated with the loss of the tumor-suppressive mir-520b/GATA6 signaling axis, thereby promoting migration and metastasis of gastric cancer cells (54). Additionally, in a recent study describing the regulation of gene expression by profiling miRNAs expression and transcription factors CREB emerged as a master regulator in multiple cancer types (55). Given the critical role of the CREB-miRNA network in influencing tumor progression by inducing transcriptional and post-transcriptional changes through multiple cellular mechanisms. It becomes imperative to comprehensively understand and utilize regulatory network information involving CREB-miRNA to design novel therapeutic strategies across several human malignancies.

## Therapeutic Targeting of CREB With Possible Implications in EMP

Given the critical role of CREB as a proto-oncogene involved in tumor initiation, progression, and metastasis, therapeutic targeting of this key transcription factor has achieved success in preclinical studies of PDAC (29). The recent findings by Kim et al. describe that activation of CREB downstream to KRAS signaling led to physical interaction with oncogenic mutant p53 (56). This interaction, in turn, activated multiple pro-tumor transcriptional programs, including FOXA1, promoting PDAC metastasis *via* the activation of the downstream WNT/β-catenin signaling axis (56). Additionally, pharmacologic inhibition using a CREB inhibitor (666–15) significantly attenuated PDAC metastasis *in vivo* (56), highlighting the essential role of CREB in the pathogenesis of this disease

Additional studies have established the involvement of the Wnt/β-Catenin signaling pathway as a major culprit of pancreatic tumorigenesis and therapeutic resistance. Wnt ligands act through autocrine or paracrine manners to bind to cognate receptors, thereby initiating a phosphorylation cascade. This permits dissociation of β-catenin degradation complex, allowing for translocation of β-catenin across the nuclear membrane (57, 58). Once inside the nucleus, β-catenin further regulates the expression of target genes, including the cAMP response element-binding protein (CBP, CREB binding protein). Similarly, Arensman et al. demonstrated that the small molecule ICG-001 binds cAMP-responsive element-binding (CREB)-binding protein (CBP) to disrupt its interaction with β-catenin and inhibit CBP function as a coactivator of Wnt/β-catenin–mediated transcription. Treatment with this inhibitor significantly improved overall survival in an *in-vivo* orthotopic xenograft model of PDAC, further establishing that disruption of CBP activity impacts PDAC tumor burden (59).

EMP in PDAC is mediated through multiple signaling pathways, among which TGF-β signaling has been shown to be the most prominent cellular pathway (60, 61). Previous studies have shown the correlation of TGF-β overexpression with poor prognosis in PDAC patients and directly associated with promoting tumor cell proliferation and invasion (62). Although it is currently unclear how CREB signaling regulates EMP in PDAC, previous studies have suggested that CREB signaling influences TGF-β signaling in pancreatic cancer cells and fibroblasts. Along with E1A binding protein, EP300, CREB can influence the EMP by regulating the E-cadherin expression (63). There are studies that established the association of CREB activity as an essential driver of tumor aggressiveness; however, there is a lack of molecular and cellular evidence exploring the regulation and activation of CREB as well as its role in influencing the EMP transcriptional program, warranting future investigations of these mechanisms in PDAC. Therefore, there are many remaining questions about the mechanisms through which CREB functions, and elucidation of this critical transcriptional regulator may have significant implications for the success of targeting strategies.

## Conclusions and Future Perspectives

The prognosis of patients with pancreatic cancer has not improved notably despite considerable research efforts. The complex interplay of signaling pathways driving EMP programs promoting therapeutic resistance and local and distal recurrence presents a significant obstacle to the current treatment regimen for this disease. Therefore, identifying new candidate molecules, such as CREB, responsible for these aberrant cellular processes, is critical for targeted therapies against the tumor heterogeneity in PDAC. Future investigations using *in vivo* manipulation of CREB expression in PDAC to better recapitulate the spontaneity and heterogeneity of human tumors may provide more robust scientific evidence of its role in regulating EMP, metastasis, and therapeutic resistance in PDAC. Importantly, understanding how transcription factors regulate EMP is still an area of intense study with emerging therapeutically relevant insights.

## Author Contributions

SM, SS, and NN were involved in the initial review design and composition. SM, SS, and NN contributed to the manuscript content and format. All authors contributed to the article and approved the submitted version.

## Funding

This work was supported by the American Cancer Society IRG 98-277-13, NIH NCI R03 CA249401, and James Esther and King Biomedical Research Program by Florida Department of Health grant 22K06 to NN.

## Author Disclaimer

The content is solely the responsibility of the authors and does not necessarily represent the official views of the NIH.

## Conflict of Interest

The authors declare that the research was conducted in the absence of any commercial or financial relationships that could be construed as a potential conflict of interest.

## Publisher’s Note

All claims expressed in this article are solely those of the authors and do not necessarily represent those of their affiliated organizations, or those of the publisher, the editors and the reviewers. Any product that may be evaluated in this article, or claim that may be made by its manufacturer, is not guaranteed or endorsed by the publisher.
